# Application and effectiveness of experiential education combined with health coaching techniques in extended care for elderly patients with dental implants

**DOI:** 10.3389/froh.2025.1538886

**Published:** 2025-06-17

**Authors:** Juan Huang, Fanghua Gong, Zhou Chen, Yali Chen, Yufei Liu

**Affiliations:** ^1^ Department of Stomatology, Hunan Provincial People's Hospital and The First Affiliated Hospital of Hunan Normal University, Changsha, Hunan, China; ^2^ Department of Nursing, Hunan Provincial People’s Hospital and The First Affiliated Hospital of Hunan Normal University, Changsha, Hunan, China; ^3^ Department of Nursing, Hunan Institute for Tuberculosis Control Hunan Chest Hospital, Changsha, Hunan, China

**Keywords:** dental implants, experiential education, health coaching techniques, geriatric care, nursing

## Abstract

**Objective:**

Experiential education (EE) and health coaching techniques (HCT) were proved to be effective in health management. This study investigated the effectiveness of EE combined with HCT in extended care for elderly patients with dental implants.

**Methods:**

A total of 90 elderly patients who received implant restoration were randomly divided into intervention group (IG) and control group (CG). CG received standard health education. IG received EE, HCT and standard health education. Periodontal conditions of peri-implants (plaque Index, PLI; sulcus bleeding index, SBI; gingival Index, GI) were assessed at 1-, 3-, and 6-months. Oral health self-efficacy and implant survival rates after 6 months were compared between two groups.

**Results:**

No significant differences in periodontal indices were observed between groups at 1 and 3 months post-intervention. After 6 months of intervention, the experimental group showed significantly superior PLI, mSBI, and GI scores relative to the control group (*P* < 0.05). IG also showed an improvement in oral health self-efficacy compared to the CG (*P* < 0.05). The implant survival rate in the IG was higher than in the CG after 6 months, but the difference was not significant.

**Conclusion:**

EE combined with HCT improves oral health self-efficacy and periodontal health in elderly patients with dental implants.

## 1 Introduction

Global aging is accelerating with the increasing life expectancy and declining fertility rates, and the aging process much faster in China than in developed countries (1, 2). According to the Fourth National Oral Health Epidemiological Survey in China (3), 81.7% of adults aged 65–74 suffer from tooth loss, driving a growing demand for dental implants—a gold-standard tooth replacement method that preserves adjacent teeth and restores natural masticatory function (4–7). While implant success rates in healthy elderly patients parallel those in younger populations (4–7), long-term maintenance remains a critical challenge. In 2018, Consensus of the International Association of Oral Implantology recommended personalized oral hygiene guidance and maintenance for implant patients, along with regular supportive therapy and monitoring (8). However, clinical practice reveals significant gaps: elderly implant patients frequently lack structured follow-up care, and oral nurses seldom engage in oral assessments and extended care for implant patients. This disconnect is particularly alarming given the age-related risks of peri-implant diseases and self-care limitations in older adults (8). A survey of elderly patients with dental implants revealed that 57% were unable to perform oral self-care (9). Therefore, innovative health education models are urgently needed.

Experiential education (EE), a transformative learning approach that bridges theoretical knowledge and practical skills through reflective practice (10), has demonstrated a significant value across various clinical practices. For instance, EE enhanced professional identity in pharmacy students (11) and improved breastfeeding competency in pregnant women (12). Additionally, health coaching techniques (HCT) is a patient-centred, goal-oriented approaches, empower individuals to achieve health goals through structured steps (contact, observation, reinforcement, clarification, assistance, inspiration, education, and guidance) (13). Internationally recognized as an advanced health management method, HCT emphasizes patient motivation and collaboration with healthcare providers to improve chronic disease management outcomes (13). Proven effective in chronic disease management (14, 15), HCT's potential remains unexplored in geriatric implant care. Given the complementary strengths of EE in skill internalization and HCT in sustained behavioural change, we postulate that integrating both approaches may synergistically enhance oral health self-efficacy and peri-implant outcomes in elderly patients. However, as far as we know, there are no intervention study have investigated the synergistic effects of EE with HCT for the extended care of elderly patients with dental implants in China.

To address this gap, this study aims to evaluate the combined effectiveness of EE with HCT on oral health self-efficacy, peri-implant periodontal conditions, and implant survival rates for patients with dental implants through a prospective study. By addressing the critical lack of evidence-based extended care protocols for aging implant populations, this study aims to provide actionable insights for optimizing postoperative management strategies in geriatric dentistry.

## 2 Methods

### 2.1 Study design and patients

This is a prospective randomized controlled trial. Elderly patients who received implants were randomly selected from April to September 2023 in the Department of Stomatology at People's Hospital of Hunan Province, Changsha, China. The inclusion criteria were: (1) age ≥60 years; (2) presence of common underlying conditions (e.g., diabetes, hypertension, cardiovascular disease) and periodontal disease were under control; (3) being able to maintain oral hygiene with good occlusion; (4) All patients received Straumann implants, and after osseointegration, each was restored with all-ceramic crowns; (5) informed consent obtained, with adequate literacy and communication skills; (6) being able to navigate WeChat independently with good compliance. Exclusion criteria included severe mental disorders or burning mouth syndrome, which could impact study results, as well as individuals with adverse habits such as malocclusion, bruxism, or night grinding. Additionally, patients unable to attend regular follow-ups or currently undergoing other interventions were excluded.

### 2.2 Sample size

According to the following formula for comparing the means of two samples:

$$n1=n2=2*{\left[\frac{(Z_{\alpha }+Z_{\beta })*\sigma }{\delta }\right]}^{2}$$

According to the study by Wenjing et al. (16), the post-intervention SBI was 1.24 ± 0.14 in the traditional education group and 1.09 ± 0.20 in the empowering health education group. In this study, the significance level (*α*) was set at 0.05, and the power of the test (1 − *β*) was set at 0.9. As a result, a sample size of 28 was required for each group. Additionally, taking into account the possible sample loss, 45 cases were included in each group, and a total of 90 patients were enrolled in the study.

### 2.3 Randomization and blinding

Patients were assigned study numbers based on the timing of their implant surgeries by the visiting nurse. Using a random number table, two digits were read each time. These digits were then sorted according to their size. Patients with the sorted odd numbers were allocated to the control group (CG), while those with the sorted even numbers were allocated to the intervention group (IG). The specific group information cannot be known until the data entry and analysis are completed.

### 2.4 Intervention

**Figure 1:**
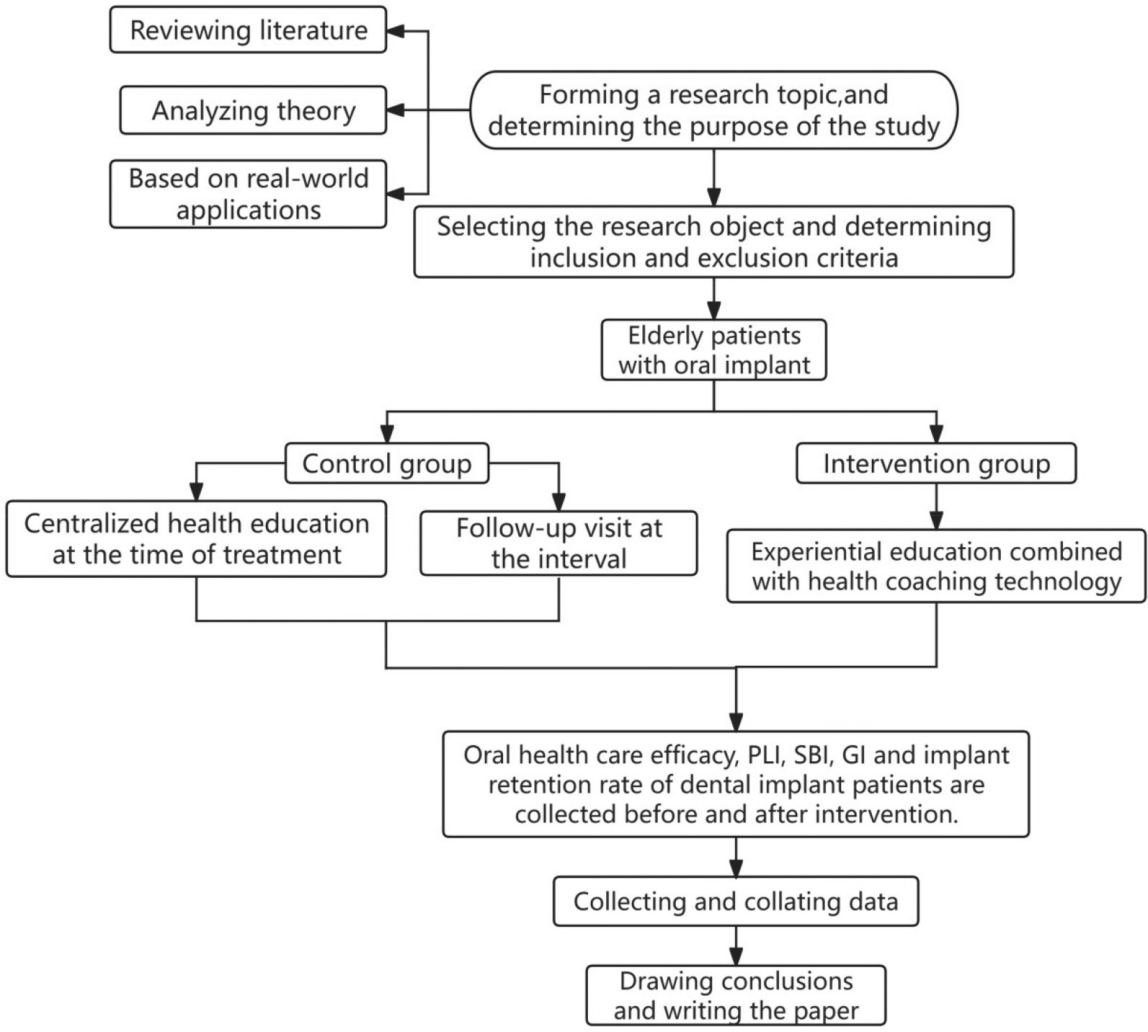
Study flowchart.

The study flowchart can be seen in Figure 1. Both patient groups received systematic health education and follow-up care after suture removal. This included educational videos on dental implant care played in the waiting room, face-to-face chairside education, distribution of educational brochures, bi-weekly telephone follow-ups during the first month post-surgery, monthly calls starting from the second month, and regular follow-up visits at 1-, 3-, and 6-months post-surgery.

In addition to the systematic health education and follow-up, the IG received an EE and HCT intervention program (EE & HCT) for 6 months. The method of EE and HCT have been demonstrated to be effective in promoting knowledge acquisition and behavior modification in previous studies (14, 15, 17). This program included: (1) Formation of an expert team consisting of two specialized oral health nurses (one for assessments and feedback, the other for tracking and observations), a specialized implant doctor, a nutritionist, and a postgraduate student for data collection and analysis. (2) Professional training in EE & HCT for the team. (3) Establishment of a WeChat group named “Implant Home” to facilitate communication and support among the expert team, patients, and their families. The intervention details were presented in Table 1.

**Table 1:** Intervention details of experiential education combined with health coaching techniques.

Process	Intervention descriptions	Goals
Contact	• Face-to-face interactions and WeChat calls and video chats. • Experiential education: watch complication cases (e.g., gingivitis, periodontitis, peri-implantitis, and implant failure) along with model demonstrations; facilitate open communication with patients, encourage sharing of experiences, and distribute educational handbooks. • Theoretical and practical education: provide lectures using PowerPoint to elucidate factors contributing to the failure of implant denture restorations in the elderly. Facilitate hands-on sessions utilizing models and educational handbooks to instruct patients in techniques such as the BASS (18) brushing method, the use of dental floss, interproximal brushes, and irrigators, as well as conducting plaque self-checks (19) and making informed dietary choices; actively encourage patient engagement and participation in these educational activities.	• Contact establishment: regularly monitor patients' oral health status through direct communication and assessment. • Sensory stimulation and risk awareness: simulate the experience of complications, enhance patients' understanding and awareness of potential risks; educate patients to identify early signs of complications, such as mucosal pain, swelling, and bleeding around implants, and prompt timely intervention. • Reinforcement of knowledge and skills: enhance experiential learning to solidify knowledge and skills; encourage patients to critically evaluate and address unhealthy oral behaviours in their daily routines, and collaborate with the expert team to develop individualized oral health management plans.
Observation	• Supervise patient-reported plaque self-checks every night.	• Evaluate adherence to health management plans, record oral health status in real time, and identify potential risks.
Reinforcement	• Encourage patients to proactively report their oral health status in WeChat groups and collaboratively formulate individualized oral health plans based on their specific conditions	• Empower patients to take responsibility of their oral health decisions, foster self-directed behaviour change, and promote sustained adherence to good oral hygiene practices.
Clarification	• Assist patients in identifying potential risks by introducing methods for plaque self-checking.	• Empower patients to assess their oral hygiene status to facilitate adjustments to their oral health plan.
Assistance	• In case of emergencies, patients can contact the expert team urgently.	• Provide instant assistance, identify contributing factors and correct detrimental habits.
Inspiration	• Encourage patients to share their difficulties and feelings with empathy.	• Provide motivation.
Education	• Provide WeChat videos, graphic and text posts, online consultations, and educational resources; give a 60-minute health lecture once a month; conduct theoretical and practical evaluations.	• Offer educational support on oral health knowledge and skills.
Guidance	• Provide timely rewards such as cash bonuses, flowers, and gifts to patients who manage their oral health well, encourage them to share their experiences.	• Guide patients to enhance their internal motivation and sense of purpose by integrating oral health goals into their daily routines.

### 2.5 Outcomes

#### 2.5.1 Oral health self-efficacy

We used the Oral Health Self-Efficacy Scale for Patients With Dental Implants developed by Rong bing et al. in 2019, with a Cronbach's *α* coefficient of 0.910 (20). This scale comprises 16 items across 3 dimensions: dental surgery, postoperative support care, and oral hygiene habits self-efficacy. It employs a 5-point Likert rating scale ranging from 1 (completely not confident) to 5 (very confident). Higher scores indicate greater self-efficacy in dental implant patients. Scores are categorized as follows: 0–60 for low levels, 60–70 for moderate levels, and 70–80 for high levels.

#### 2.5.2 Peri-implant periodontal conditions

Peri-implant periodontal condition was assessed by a combination of probe stroking and visual inspection by a dental professional (21). The indexes included plaque index (PLI), sulcus bleeding index (SBI), and gingival index (GI).

PLI: Proposed by Silness and Loe, this index assesses the amount and thickness of dental plaque by gently scraping the tooth surface with a probe. Each tooth's four surfaces (mesial, central, and distal buccal, plus lingual) are examined. Tooth score is the average of four—surface scores; individual score was the average of all examined teeth scores. A score of 0 indicates no plaque; (1) indicates a thin layer of plaque; (2) indicates a moderate amount of plaque or calculus visible to the naked eye; (3) indicates abundant plaque or severe calculus.

SBI: Proposed by Mombelli, this index involves probing around the soft tissues of the implant with a periodontal probe 1 mm subgingivally for 30 s. Bleeding responses were recorded: 0 (none), 1 (pinpoint), 2 (linear), 3 (spontaneous).

GI: This index assesses changes in gingival colour, texture, and bleeding tendency through probing with a blunt—ended periodontal probe. Each tooth's four surfaces are examined. Each tooth's score is the average of its four—surface scores, and the individual score is the average of all examined teeth scores. Scores were recorded: 0 = healthy gums; 1 = mild gingivitis (gums do not bleed but show slight colour change and mild swelling); 2 = moderate gingivitis (gums bleed on probing, redness, swelling); 3 = severe gingivitis (gums bleed spontaneously, significant redness, swelling).

#### 2.5.3 Implant survival rates

Implant survival rates were calculated by dividing the number of retained implants by the total number of implants (21).

### 2.6 Data collection

The two groups were assessed with questionnaires anonymously at baseline and at 1-, 3-, and 6-month post-intervention, including baseline characteristics and Oral Health Self-Efficacy Scale for PatientsWith Dental Implants. Peri-implant periodontal conditions and implant survival rates were recorded. Researchers offered explanations for any queries regarding the questionnaire content if necessary. Upon completion, the researchers reviewed and collected the questionnaires on-site. After the intervention, the control group withdrew 1 case (loss to follow-up) and finally recovered 89 cases.

### 2.7 Statistical analyses

Data analysis was performed using SPSS version 25.0. Baseline data for the two groups of dental implant patients were treated as categorical variables and analysed using the Chi-square test. Oral health self-efficacy scores were reported as means ± standard deviations, with normality and homogeneity of variance assumptions. Between-group differences in self-efficacy were assessed using the independent samples *t*-test. The PLI, SBI, and GI were treated as ordinal data and analysed using the Mann–Whitney *U* test for independent samples. Implant survival rates, considered as count data, were analysed with the corrected Chi-square test. A significance level of *P* < 0.05 was used for all statistical tests.

## 3 Results

### 3.1 Baseline characteristics

A total of 89 elderly dental patients were included in this study. The baseline characteristics of patients were presented in Table 2. No significant differences were observed between the IG and CG regarding age, sex, residence, educational level, average monthly income, and the number of dental implants (*P* > 0.05).

**Table 2:** Baseline characteristics (*N* = 89).

Variables	Categories	*N*	IG	CG	*χ* ^2^	*P*
Age (years)	60–70	52	25	27	0.309	0.578
	>70	37	20	17		
Sex	Male	58	28	30	0.348	0.555
	Female	31	17	14		
Residence	Urban	54	28	26	0.091	0.762
	Rural	35	17	18		
Educational level	Middle school and below	30	16	14	0.296	0.862
	High school/technical secondary school	11	6	5		
	College and above	48	23	25		
Average monthly income (CNY)	<2,000	26	14	12	0.286	0.867
	2,000–4,000	44	21	23		
	>4,000	19	10	9		
Dental implants	Anterior teeth	49	25	24	0.001	0.969
	Posterior teeth	111	57	54		

### 3.2 Oral health self-efficacy

Before the intervention, there were no statistically significant differences in total self-efficacy scores and across all three dimensions between the IG and the CG (*P* > 0.05). However, at six months post-intervention, the IG demonstrated significantly higher scores in all dimensions compared to the CG (*P* < 0.05), as detailed in Table 3.

**Table 3:** Oral health self-efficacy between groups before and after intervention.

Time	Groups	Dental surgery	Postoperative support care	Oral hygiene habits	Total scores
Before intervention	CG	21.02 ± 2.13	21.63 ± 3.37	23.32 ± 3.98	65.97 ± 8.42
	IG	21.13 ± 2.07	21.48 ± 3.39	23.22 ± 4.05	65.83 ± 8.45
	*t*	−0.183	0.093	0.228	0.123
	*P*	0.855	0.926	0.820	0.902
After intervention	CG	21.87 ± 2.03	22.03 ± 3.31	24.99 ± 3.96	68.89 ± 8.15
	IG	23.06 ± 2.09	23.19 ± 3.35	27.94 ± 4.06	74.19 ± 8.34
	*t*	−2.184	−2.162	−4.226	−4.753
	*P*	**0.032**	**0.033**	**<0.001**	**<0.001**

*P* value in bold letter is statistically significant.

IC, intervention group; CG, control group.

### 3.3 Peri-implant periodontal condition

At 1- and 3-months post-intervention, there were no statistically significant differences in PLI, SBI, and GI between IG and the CG (*P* > 0.05). However, at 6-months post-intervention, the IG exhibited significantly lower PLI, SBI, and GI scores compared to the CG, with these differences being statistically significant (*P* < 0.05), as presented in Table 4.

**Table 4:** Peri-implant periodontal conditions between groups.

Outcomes	1 month		3 months		6 months	
	CG	IG	CG	IG	CG	IG
PLI						
Level 0	34	37	28	32	17	29
Level 1	8	7	8	8	11	12
Level 2	2	1	6	5	10	3
Level 3	0	0	2	0	6	1
*Z*value	−0.611		−0.902		−2.984	
*P*	0.541		0.367		**0.003**	
SBI						
Level 0	31	33	26	30	16	27
Level 1	9	10	10	11	11	14
Level 2	4	2	6	4	10	3
Level 3	0	0	2	0	7	1
*Z*value	−0.412		−0.985		−2.836	
*P*	0.680		0.325		**0.005**	
GI						
Level 0	32	35	27	31	16	28
Level 1	8	7	7	7	10	11
Level 2	4	3	7	6	11	5
Level 3	0	0	3	1	7	1
*Z*value	−0.566		−0.878		−2.983	
*P*	0.571		0.380		**0.003**	

*P* value in bold letter is statistically significant.

IC, intervention group; CG, control group.

### 3.4 Implant survival rates

After six months, the IG (*n* = 45) had 82 dental implants, with 1 failure, resulting in an implant survival rate of 98.78%. The CG (*n* = 44) had 78 implants, with 2 failures, resulting in an implant survival rate of 97.44%. All other implants remained stable with no loosening and good functional recovery. The implant survival rates between the two groups showed no statistically significant difference (*P* > 0.05), as detailed in Table 5.

**Table 5:** Implant survival rates between groups.

Groups	Implants condition		χ^2^	*P*
	Survived	Failed		
IG	82	1	0.001	0.974
CG	78	2		

IC, intervention group; CG, control group.

## 4 Discussion

This study applied experiential education combined with health coaching techniques in the extended care of elderly patients with dental implants. Our findings indicated at six months post-intervention, the oral health self-efficacy scores for patients with dental implants in the intervention group significantly improved compared to the control group. Additionally, at six months post-intervention, the peri-implant periodontal condition of the intervention group was significantly better than that of the control group, including PLI, SBI, GI. Although the implant survival rate of the intervention group was higher than that of the control group at six months post-intervention, the difference was not statistically significant.

In this study, baseline oral health self-efficacy scores in elderly dental implant patients were moderate to low in both groups. At six months post-intervention, the IG demonstrated high overall self-efficacy, with scores in dental surgery, postoperative support care and oral hygiene habits significantly higher than those in the control group. This is consistent with the findings of Johansson et al. that the oral health self-efficacy of elderly residents in nursing homes improved with the implementation of HCT (22). A plausible explanation is the involvement of dental professionals in the entire process and the integration of auditory, visual, and practical content by applying EE and HCT, thereby fully engaging patients' enthusiasm and initiative (23). This comprehensive approach significantly enhances the educational impact and, consequently, improves oral health self-efficacy in the intervention group (17). Additionally, the expert team, family members, and fellow patients throughout the entire various methods, including chairside face-to-face health education, health lectures with practical demonstrations, and communication via phone, WeChat video, and voice calls, not only helped patients acquire oral health behaviours but also boosted their confidence, ultimately enhancing the oral health self-efficacy of patients in the intervention group (14, 23).

The peri-implant periodontal condition of patients in the intervention group showed significant improvement at six months post-intervention in our study. Experiential education, which integrates theory and practical training, along with the “partnership” approach of health coaching, allows patients to interact and negotiate with health coaches, jointly setting oral health goals and specific plans. By dynamically understanding patients' oral health status and providing timely guidance and assistance, patients' knowledge and skills in oral health care are greatly enhanced. This, in turn, promotes the formation and maintenance of good oral health behaviours, aiding in the active control of dental plaque and improving peri-implant periodontal conditions in elderly dental implant patients. Research has confirmed peri-implantitis is the most common cause of implant failure and the treatment of peri-implant inflammation is costly and difficult (24). A survey indicated that the prevalence of peri-implantitis is as high as 22%. Dental plaque, as the primary cause of periodontitis, colonizes around implants, leading to gingival swelling, bleeding during brushing, and eventually peri-implantitis (25). Angelov N et al. noted that age is a risk factor for periodontal disease (26). Comprehensive and scientific oral health education helps elderly implant patients develop good oral health behaviours, which is crucial for controlling dental plaque and preventing complications (27). Effective oral health management is essential for the long-term success of implant prostheses. Studies by Moes Shinta L et al. demonstrated that situational experiential education applied to pregnant women effectively improved their breastfeeding ability and parenting efficacy (12). A systematic review by Barnet-Hepples T et al. concluded that health coaching in adults with chronic non-cancer pain significantly improved physical activity, disability, and pain management (28). These findings suggest that experiential education and health coaching are effective management approaches.

Regarding the survival rate of implants, there were three implant failures documented and the difference was not statistically significant between groups in our study. Two cases involved patients who continued smoking over 20 cigarettes daily despite medical advice, and one case involved a diabetic patient with poor oral hygiene, leading to deteriorated glycaemic control and subsequent implant failure. The remaining implants demonstrated stability with satisfactory osteointegration. Previous studies have identified smoking as an independent risk factor for peri-implantitis, which ultimately leads to implant loosening and failure (29). A meta-analysis by Shang et al. concluded that type 2 diabetes elevates the risk of peri-implantitis by 3.39 times compared to healthy individuals by inhibiting the proliferation and differentiation of osteoblasts, disrupting the oral microenvironment, stimulating inflammation, and compromising oral health (30). Therefore, stringent glycaemic control is imperative for diabetic patients to ensure implant success. Schimmel et al. reported that patients with poor oral hygiene are more likely to develop peri-implantitis compared to those maintaining adequate oral hygiene (31). Providing systematic, continuous, and high-quality oral health management by a team of experts can enhance internal motivation and goal-setting among patients (15). Integrating oral health goals into daily routines may further promote and sustain good oral health behaviours. The statistically insignificant difference between groups, however, may be due to the limited sample size or the short observation period. Nonetheless, the intervention group exhibited an improved survival rate, indicating the potential efficacy of the intervention. Future research involving larger sample sizes and extended intervention periods is warranted to assess the impact of this intervention more comprehensively on implant survival rates.

### 4.1 Strengths and limitations

Our study has several limitations that warrant consideration. First, the study was conducted at a single center, which limits the generalizability of the findings due to the restricted sample source and potential lack of representativeness of a broader population. Second, the limited duration of the study might have failed to capture long-term effects, potentially compromising the generalizability and long-term validity of the conclusions. Future research should address these limitations by refining the intervention protocol, extending the intervention period, and expanding the sampling scope to enhance the robustness of the results. However, our study leveraged the advantages of combining experiential education and health coaching techniques to develop an intervention model tailored for implant patients. To our knowledge, no previous research has applied these techniques in the context of extended care for elderly oral implant patients, thereby providing a novel reference for clinical nursing practices. Additionally, we explored the impact of this intervention on various outcomes, including oral health self-efficacy, peri-implant periodontal conditions, and implant survival rates, thereby offering further evidence to support clinical care for implant patients.

## 5 Conclusion

The combination of experiential education and health coaching techniques for the extended care of elderly oral implant patients significantly enhances their oral health self-efficacy, facilitates good oral hygiene practices, and improves peri-implant periodontal health. The impact on implants survival rate should be explored further.

## Data Availability

The raw data supporting the conclusions of this article will be made available by the authors, without undue reservation.
